# Economic incentives to use fertilizer on maize under differing agro-ecological conditions in Burkina Faso

**DOI:** 10.1007/s12571-018-0842-z

**Published:** 2018-09-15

**Authors:** Veronique Theriault, Melinda Smale, Hamza Haider

**Affiliations:** 1 Department of Agriculture, Food and Resource Economics, Michigan State University, East Lansing, MI 48824, USA

**Keywords:** Yield response to nitrogen, Value-cost ratios, Subsidy, Agro-ecologies, West Africa

## Abstract

Increasing agricultural productivity while protecting natural resources depends on proper understanding of farmers’ incentives to use intensification strategies, including fertilizer. Using a large-scale household dataset collected in rural Burkina Faso, we examined how the response of maize yield to fertilizer, and thus the economic incentives for its use, varied according to agro-ecological conditions. We employed a Control Function Approach with Correlated Random Effects in order to test and control for endogeneity of fertilizer use, measuring agro-ecological conditions at several scales. We investigated the profitability of fertilizer use with value-cost ratios. We found that productivity and marginal effects of fertilizer differ significantly according to agro-ecological conditions. Micro-variation appeared to be more critical than the definition of agro-ecological zone. Burkinabe soils are severely degraded and would benefit from greater application of fertilizer. However, at full market prices, fertilizer use was unprofitable. Though it was profitable with subsidized prices, transaction costs diminish the benefits of the subsidy. Profitability of fertilizer use with maize varied across agro-ecological conditions, even for field plots located in the same agro-ecological zone. Our results confirm that policy makers need to be cautious when generalizing across regions or drawing policy recommendations from a single agro-ecological zone because crop responses and economic incentives vary widely.

**JEL classification** O13 . Q12 . Q16 . Q18

## 1 Introduction

Achieving food security in Sub-Saharan Africa depends crucially on raising the productivity of smallholder farmers – the cornerstone of most agricultural economies in that region. Designing suitable policies to boost productivity while protecting natural resources depends on proper understanding of farmers’ incentives to use intensification strategies, including rates of fertilizer. Underlying agro-ecological conditions can shape the response of crop yield to fertilizer, which in turn affects economic incentives to use this relatively costly input. Yet, most of the agricultural policies to promote fertilizer use, such as input subsidy programs, are implemented at the national scale with the same recommendations for all. Such “blanket” recommendations have long been criticized because they ignore the heterogeneity of rainfall and soil fertility across agro-ecologies (for recent examples, see Snapp et al. 2003 and Kaizzi et al. 2017).

Poor drainage and limited availability of moisture constrain many of the soils in Sub-Saharan Africa, along with spatial and temporal concentration of rainfall. The soils in the Sahel and Savanna of West Africa are old and low in soil organic matter, with low capacity to retain nutrients, while this region is also the most densely populated in the continent (Jones et al. 2013). The climatic vulnerability of West Africa, aggravated by high rates of population growth, has prompted major efforts by governments and farmers themselves to intensify crop production sustainably (Reij et al. 2009). Recent analysis of satellite imagery confirms that between 2000 and 2013, the progression of agriculture has accelerated in Burkina Faso. Wooded savanna in the Sudanian zone of the country has been replaced entirely by rainfed crops, with natural landscapes throughout ceding to a mosaic of crops and fallows (CILSS 2016). To enhance crop production in Burkina Faso, there will be no other option than intensification.

As in most nations of Sub-Saharan Africa, the national agricultural research system in Burkina Faso formulated fertilizer recommendations during the 1970s and 1980s, but these did not, and still do not, take differing rainfall regimes or other aspects of growing conditions into account. Although fertilizer is more widely available today than in the past, effective demand for inputs is often unreliable since it depends closely on farmer access to input and output markets. Vanlauwe et al. (2010) and Kihara et al. (2016) explain that the heterogeneity of overall agro-ecological and soil conditions at regional, national, and local scales has led to a diversity of farming systems, cropping patterns, soil management considerations, and input markets. This diversity is likely to entail highly variable economic incentives for smallholder farmers.

These observations drive our central hypotheses that the response of maize yield to fertilizer in Burkina Faso, and thus the economic incentives for its use, vary by agro-ecological conditions. We tested these hypotheses by estimating a maize yield response function at the field plot level with data collected during three cropping seasons (2009/10, 2010/11 and 2011/12) under the Continuous Farm Household Survey combined with rainfall and soil data from the National Oceanic and Atmospheric Administration’s Climate Prediction Center and the European Union’s Soil Atlas of Africa. We tested and controlled for endogeneity of fertilizer with a Control Function Approach (CFA), employing Correlated Random Effects (CRE) to address time-invariant unobserved effects. We compared the robustness of the estimated marginal product of fertilizer while testing the effects of different sets of agro-ecological conditions across econometric models. The richness of the dataset allowed us to study three layers of agro-ecological conditions: 1) agro-ecological zones; 2) rainfall and soil types at the village level; and 3) land quality at the field plot level, as indicated by the presence of agroforestry, fallow, soil and water conservation structures, and topography. We then investigated the profitability of fertilizer use by calculating the marginal and average value-cost ratios based on the estimated coefficients under different price scenarios.

There is an extremely sparse literature about the yield response of maize to fertilizer in West Africa based on analysis of data collected from farm households. Yet, analyses based on farm household data are fundamental for understanding how factors outside the control of agronomic field experiments affect economic incentives for fertilizer use by smallholder farmers. Yield response estimates are also essential for predicting the productivity impacts of fertilizer subsidies, which are employed as a policy tool by many African governments (Jayne et al. 2018). In recent years, numerous analyses of maize yield response based on farm household data have been conducted in the Eastern and Southern regions of Africa (e.g., Marenya and Barrett 2009; Sheahan et al. 2013; Xu et al. 2009 and Burke et al. 2017). However, farming context and agro-ecological conditions differ enormously among these regions and the West African Sahel. We have found maize yield response models by Ragasa and Chapoto (2017) in Ghana and Koussoube and Nauges (2017) in Burkina Faso, but neither of these were able to control for unobserved, time-invariant heterogeneity due to the cross-sectional nature of their studies. Decades ago, Henao et al. (1992), Kouka et al. (1995) and others estimated agronomic optima using trial data from northern Ghana and Mali. A recent compilation of studies reports findings of agronomic research on the optimization of fertilizer use across countries of Sub-Saharan Africa, including Burkina Faso (Wortmann and Sones 2017). In a study comparing maize yield response to various rates of nitrogen fertilizer over 940 demonstration sites in Eastern and Southern Africa, Jama et al. (2017) found that applications at 50% or more of recommended rates ensured profitability. However, methods applied in agronomic studies are not comparable with ours because they are based on field experiment data instead of household survey data.

Thus, our study makes key contributions to the literature. First, ours is the only maize yield response model, we are aware of, that is estimated using panel data collected from farm households in the West African Sahel. Second, we combine information from the panel with rainfall and soils data in order to test the effects of agro-ecological conditions at three scales of analysis. Only a small subgroup of studies mentioned above have tested effects of macro-scale agro-ecological variation on maize yield response to fertilizer and profitability. Finally, we add transaction costs to the profitability analysis, bringing new insights regarding a fertilizer policy based on uniform national recommendations.

## 2 Farming context in Burkina Faso

About three-quarters of the Burkinabe population lives in rural areas and depends on agriculture as its main source of livelihood (MAFAP 2013). The traditional diet in rural areas has been based on sorghum and pearl millet as starchy staples, but maize has gained in popularity in recent years. From 2005 to 07 to 2011–13, the daily per capita consumption of maize in kilocalories rose by 26% compared to a reduction of 3 and 17% for sorghum and pearl millet, respectively (FAOSTAT 2018). Maize now ranks second in terms of production and value of production after sorghum (FAOSTAT 2018), and has become a strategic crop for food security in Burkina Faso. About one quarter of Burkinabe households have poor or limited food consumption (WFP 2014). A key strategy to improve food access is to increase agricultural productivity and income, especially for staple food crops, such as maize.

The Burkinabe land cover has changed drastically over the last decades. In 1975, about 15% of the land was under rainfed agriculture, compared to 39% in 2013, representing a 160% change in less than 40 years (CILSS 2016). Maize is among the crops that has seen the most significant increases in cultivated areas. From 1970 to 1974 to 2009–2013, cultivated areas of maize increased from 95,000 ha to 775,000 ha. Although maize yields have increased over the last decades, they still remain low with an average of 1.6 t per hectare (FAO 2015). Most of the increase in maize production has come from an expansion in arable land rather than through cropping intensity. Commercial fertilizer markets remain poorly developed (Theriault and Tschirley 2014) and overall use rates on dryland cereal crops, including maize, are but a fraction of the 50 kg/ha goal stated in the Abuja Declaration.

The use of inorganic fertilizers is the most commonly adopted practice to improve productivity in the West African Sahel, despite efforts to encourage the use of complementary practices designed to better manage soils and water or to amend soils. This region is characterized by rainfall that is infrequent but can be heavy, accompanied by runoff and soil erosion. That is why farmers and research programs in this region have developed soil and water conservation structures to retain moisture and nutrients, with the ultimate goal of sustainably increasing productivity (Reij et al. 2009; GIZ 2012; CILSS 2016).

An abundant literature exists on the positive impact of inorganic fertilizers on crop production, but it has often come at the expense of state-managed subsidy schemes, which in some instances are of questionable social return. For example, fertilizer use (i.e. nitrogen) on maize is profitable at full market prices in Ghana (Ragasa and Chapoto 2017), but not profitable without major subsidies in Zambia (Burke et al. 2017). In many African countries, a large share of the agricultural budget has been allocated to subsidies on inorganic fertilizers as a way to boost production (Jayne et al. 2018). This is also the case in Burkina Faso, where the government has implemented a program to facilitate access to fertilizer.

Specifically, the program provides financial support to the local cotton companies to purchase and distribute fertilizer on credit to cotton farmer cooperatives and subsidizes fertilizer for staple crops, such as maize and irrigated rice (Theriault and Serra 2014; Ouedraogo 2016). Subsidized 50 kg bags of NPK and urea are available only for those targeted crops (Ouedraogo 2016). Although the official subsidy rate is 50%, subsidized fertilizers are approximately only a quarter cheaper than those purchased at full market value because of high transaction costs (Holtzman et al. 2013). Maize farmers who also grow cotton have an assured access to subsidized fertilizer through their cooperatives, while maize farmers who grow no cotton have access only through the commercial market (Ouedraogo 2016). Given the government’s limited budget, not all maize farmers have access to subsidized fertilizer in any given year. Wanzala-Mlobela et al. (2013) estimated that subsidized fertilizer accounts for approximately 17% of total fertilizer consumption in Burkina Faso. Ouedraogo (2016) explained how subsidized fertilizer is provided to maize farmers who do not grow cotton. First, all villages within each commune are classified into three groups throughout the country. Second, each group participates in the fertilizer subsidy program sequentially. For example, the first group has exclusive access to subsidized fertilizer in the first year, and in the subsequent year, only group 2 has access. Third, within each village, eligible beneficiaries are identified by an official committee, with preferences given to those that apply good agricultural practices, such as manure and soil and water conservation techniques (see Ouedraogo (2016) and Wanzala-Mlobela et al. (2013) for additional details on the fertilizer value chain and subsidy program in Burkina Faso).

Officially, agro-ecological zones in Burkina Faso are constructed solely on the basis of rainfall isohyet, consisting of Sahelian, Sudano-Sahelian and Sudanian zones (Bainville 2016; De Longueville et al. 2016). The Sahelian zone has low and erratic rainfall, averaging less than 600 mm annually. Pearl millet and sorghum are the principal subsistence crops. Needing a minimum of 600 mm of rainfall per year, maize is not a crop that is well-adapted to the Sahelian zone (CIRAD/GRET 2012). In the Sudano-Sahelian zone, average annual rainfall oscillates between 600 mm and 900 mm. With the additional rainfall, the Sudano-Sahelian zone is known for its production of maize and groundnut, as well as pearl millet and sorghum. Precipitation is highest in the Sudanian zone, with an average of 900 mm to 1200 per year. The Sudanian zone is the most suitable for agriculture. Cotton and cereal fields, including pearl millet, sorghum, and maize, are all part of its landscape. Across the entire country, the rainy season lasts from three to six months, with the longest season in the Sudanian zone, and the shortest one in the Sahelian zone.

Ten different types of soils cover Burkina Faso but two-thirds of the area has soils that are iron-rich and low in organic matter content. Extensive areas of Plinthosols (i.e. iron-rich), occur in all zones (Jones et al. 2013; ESDAC 2014; FAO 2015). Plinthosols are naturally poor in fertility and hardening occurs upon repeated dry and wet conditions (i.e. rainfall seasonality). The adoption of soil and water conservation practices is strongly encouraged to reduce erosion and ease farming activities on those soils. As rainfall increases, clay-rich soils, such as Lixisols, appear in the southern part of the country (ESDAC 2014). Sandy soils (i.e. Arenosols), which have low water and nutrient retention capacity, are mostly found in the Sahelian zone (FAO 2015).

## 3 Methodology

### 3.1 Econometric strategy

Past literature on crop yield response to fertilizer, much of which involved agronomic analysis of trial data, demonstrates concern for the mathematical specification of the production function because of the implied physical relationship among inputs (e.g., Chambers and Lichtenberg 1996; Guan et al. 2006). In the uncontrolled environment of household farm production, where a large set of covariates must be considered, highly flexible forms (e.g., full quadratic or translog) become computationally infeasible. Most recent analyses of maize yield response to fertilizer in sub-Saharan Africa apply variations on quadratic models. Our functional form most closely resembles that of Sheahan et al. (2013), Ragasa and Chapoto (2017), and Burke et al. (2017), who included the quadratic term for nitrogen and interaction terms for main hypotheses of interest. In addition to a quadratic term for nitrogen, we specify interactions of nitrogen use with agro-ecological conditions. Since we are interested in understanding how agro-ecological conditions affect fertilizer use and profitability, we specified a model that includes interactions between nitrogen application rate and agro-ecological conditions at three different scales (i.e. agro-ecological zones, rainfall and soil types at the village-level, and land quality at the field plot-level).

We start with the premise that yield (Y) on maize plot i from household j in time t is a function of:

(1) $$Y_{ijt}=\alpha_{0}+\alpha_{1}N_{ijt}+\alpha_{2}{AEC}_{ijt}+\alpha_{3}X_{ijt}+\beta_{1}N_{ijt}^{2}+\beta_{2}N_{ijt}^{*}AEC_{ijt}+U_{ijt},$$

Where N_ijt_ is the nitrogen application rate, AEC_ijt_ are the agro-ecological conditions, X_ijt_ is a vector of other covariates, and *α* and β represent the parameter estimates. The error term U_ijt_ is composed of three parts: V_ijt_, E_ijt_, and C_ij_. Where E_ijt_ is the random error term, V_ijt_ are unobserved time-variant characteristics, and C_ij_ are unobservable time-invariant characteristics.

We employ a Control Function Approach with Correlated Random Effects to estimate a maize yield response to fertilizer. A Control Function Approach (CFA) is preferred to a standard instrument variable (IV) method, such as 2SLS, since it enables us to address with one instrumental variable both unobserved heterogeneity in fertilizer use and endogeneity that can result from the squared and interaction terms (Wooldridge 2010). In this case, the CFA estimator is more precise than the 2SLS estimator (Wooldridge 2007). In the first stage of the CFA, the nitrogen application rate is regressed on the instrument and all other explanatory variables using OLS. In the second stage, we used the predicted residual of the first stage as an explanatory variable to control for possible endogeneity (details included in supplementary material).

There are plausible reasons to expect violation of the exogeneity assumption, through the correlation of fertilizer application with unobserved time-variant and invariant characteristics and the random error term. Not controlling for potential endogeneity issues could lead to biased estimates of the effect of nitrogen application on maize yields.

First, a potential source of endogeneity can arise from the correlation between nitrogen application and time-variant omitted characteristics (V_ijt_), such as seasonal agronomic conditions and managerial skills. To control for unobserved timevariant omitted characteristics, we included rainfall averages and variability at the village level, presence of anti-erosion structures (Soil Water Conservation, SWC), agroforestry, fallowing, and plot manager characteristics.

Second, we tested and controlled for potential causal endogeneity-correlation between fertilizer use and random error term (E_ijt_), by employing an instrumental variable technique. This consisted of regressing the nitrogen application rate on the instrumental variable as well as all other explanatory variables, the first stage of the Control Function Approach. To be valid, the instrument must be sufficiently correlated with fertilizer application (inclusion restriction) and uncorrelated with the error term (exclusion restriction). The validity of the instrument is verified through the F-statistic of the regression along with the significance of the instrument coefficient. After testing several of the instruments used in previous research on the topic, our strongest was the proportion of households in the commune that belong to cotton cooperatives.^1^ Since commercial fertilizer markets are still underdeveloped in Burkina Faso, cotton cooperatives remain the primary source of access to fertilizer (Theriault and Tschirley 2014), but membership is not correlated with soil characteristics. In Burkina Faso, cotton is cultivated in rotation with dryland cereals, such as maize. Some fertilizer provided on credit by the local cotton companies is diverted from cotton to maize fields. In an effort to reduce fertilizer diversion, which is detrimental to cotton productivity, local cotton companies have recently provided fertilizer on credit for both cotton and maize crops (Theriault and Serra 2014).

Third, Correlated Random Effects (CRE), also known as the Mundlak-Chamberlain device, controls for omitted timeinvariant unobservable characteristics (C_ij_), such as ethnicity of the household head or intrinsic quality of the land, that may be correlated with both yield and fertilizer use. The omission of land quality indicators could generate a positive bias in the effect of nitrogen on maize yields. We also controlled for fixed agronomic conditions by including agro-ecological zones and soil types. Unlike the use of fixed effects models, the Mundlak-Chamberlain device allows us to recover the coefficients of important time-invariant explanatory variables. The household unobserved time-invariant effects are correlated with the observed covariates, through the projection of those effects on the time average of covariates. Operationally, the approach involves adding time averages of household explanatory variables that vary over the years of the survey to the regression.

All standard errors were bootstrapped. Bootstrapping takes into account that maize yields of plots belonging to the same household may be correlated and also deals with the inclusion of the predicted regressor (control function-fitted residual from the first stage) in the second stage regression.

### 3.2 Profitability

To examine the profitability of fertilizer use, we first obtained the marginal product of N by taking the partial derivative of expected yields conditional on the set of covariates with respect to N in the regression equation. To find the optimal quantity of nitrogen to apply from an agronomic view point, we set the derivative equal to zero and solved for “N”. Then, we investigated profitability through examining the economic optimum, marginal and average value/cost ratios. The economic optimum indicates the N rate at which the last increment of N returns an increase in yield large enough to pay its cost. The marginal value/cost ratio (MVCR^2^) measures the ratio of the incremental value in maize output per kg of N to the price per kg of N.^3^ The general rule is that profit is maximized by applying the quantity of nitrogen at which marginal revenues equal marginal costs, or MVCR equals one. However, in a high-risk production environment, such as that of dryland farming in Burkina Faso, a ratio of two has been considered more reasonable (Ragasa and Chapoto 2017; Sheahan et al. 2013; Xu et al. 2009).

We computed MVCRs under various fertilizer costs and farm gate prices for maize. An average low, mean, and high price value for maize was computed using monthly farm gate prices across the three crop years (INERA 2013). We also considered three different fertilizer costs: market price, official subsidized price, and transacted subsidized price. The subsidized fertilizer prices for urea and NPK are set at 270 FCFA/kg and 250 FCFA/kg, which is 50% below market prices (MAFAP 2013). High transactions costs, due in part to poor road infrastructure and illicit tax collection, reduce the effective subsidy by 28 and 23% of the market price for urea and NPK, respectively, compared to the official 50% price reduction (Holtzman et al. 2013). Differences between the official and transacted subsidized fertilizer prices have also been found in Mali and Nigeria (Theriault et al. 2018; Liverpool-Tasie 2014).

The profitability incentive to use fertilizer has also been examined frequently with the average value cost ratio (AVCR^4^) (Burke et al. 2017; Xu et al. 2009; Marenya and Barrett 2009; Morris et al. 2007). An expected AVCR greater than one would indicate that risk-neutral farmers would increase their income by using more fertilizer. However, for risk-averse farmers, profitability has been considered low if the AVCR is less than two (Morris et al. 2007). When production or price risk is high, an AVCR ratio of three to four has been considered necessary to ensure profitability (Kelly 2005). In countries such as Burkina Faso, where maize production is entirely rainfed, we propose a minimum AVCR of three.

### 3.3 Data

Crop production, plot and household data were drawn from the Continuous Farm Household Survey (*Enquête Permanente Agricole* (EPA)) of Burkina Faso. The EPA has been implemented by the General Research and Sectoral Statistics Department (*Direction Générale des Études et des Statistiques Sectorielles*) of the Ministry of Agriculture and Food Security (*Ministère de l’Agriculture et de la Sécurité alimentaire*) for many years. The EPA serves as a tool to monitor food security and implement agricultural policies in Burkina Faso. The sampling frame for the EPA is based on the 2006 Population Census and is nationally representative. It covers household farms located in 826 villages across all 45 provinces. The EPA provides information on production, area, yield, and farm input use for rainfed crops, including maize, as well as general information on livestock holdings, income, and expenditures. We utilized data for the 2009/10, 2010/11 and 2011/12 cropping seasons, which are the last years for which fully cleaned data are available. After excluding households that did not cultivate maize, we had 2321 households (out of 2700) and 6701 maize plots.

To examine agro-ecological conditions at different scales, we linked GPS coordinates for the village of each surveyed household to rainfall data from the National Oceanic and Atmospheric Administration’s Climate Prediction Center and to soils information from the European Union’s Soil Atlas of Africa. Each survey village was assigned to an official agro-ecological zone based on its average rainfall history over the last decade. Virtually all maize plots were located in the Sudano-Sahelian (between the 600 mm isohyet and 900 mm isohyet) and Sudanian (above 900 mm isohyet) zones.^5^ The annual rainfall and coefficients of variation in total annual rainfall at the village level over the last three years were also computed. Following the Harmonized World Soil Database (Jones et al. 2013), we used the GPS coordinates to identify the different soil types in our sample and classify them, based on their suitability for maize production, into three groups: 1) excellent (Cambisols, Luvisols, and Nitisols); 2) good (Vertisols and Regosols); 3) poor and marginal soils (Arenosols, Leptosols, Lixisols, Plinthosols, and Planosols). Details on soil types can be found in Jones et al. (2013). We used the presence of agroforestry and soil and water conservation practices, duration of last fallow, and topography as indicators of land quality at the plot-level, as reported in the EPA dataset. Agroforestry, fallow, and soil and water conservation practices all play important functions in restoring soil fertility and topography can explain differences in land quality.

### 3.4 Variables

Table 1 provides the definitions and summary statistics of variables included in the yield response function. In addition to testing various agro-ecological factors measured at three scales of analysis (field plot, village, zone), we utilized the EPA dataset to control for a wide range of production inputs, plot, plot manager, and household characteristics. Other production inputs and plot manager characteristics have been mostly overlooked in previous studies.

**Table 1:** Variable definitions and summary statistics

Variable	Definition	Mean	Std. Dev.
Yield_ijt_	Maize yield (kg/ha)	1256	756
N_ijt_	Nitrogen application (nutrient kg/ha)	15.60	29.03
Agro-ecological conditions			
SWC_ijt_	1 = soil and water conservation structure on the plot; 0 = no	0.126	0.332
Fallow_ijt_	Number of years since the plot was left fallow (years)	18.91	15.60
Agroforestry_ijt_	1 = trees on the plot; 0 = no	0.590	0.491
Lowland_ijt_	1 = lowland plot; 0 = no	0.055	0.228
Slope_ijt_	1 = plot with a steep slope; 0 = no	0.062	0.241
Rain_jt_	Total rainfall in the village (mm)	955	143
CV_jt_	Coefficient of variation of rainfall in the village over the last three years (mm)	0.090	0.043
Excellent soils_j_	1 = Cambisols, Luvisols, and Nitisols; 0 = no	0.218	0.413
Good soils_j_	1 = Vertisols and Regosols; 0 = no	0.094	0.293
Sudano-sahelian_j_	1 = Sudano-sahelian zone; 0 = no	0.559	0.496
Plot characteristics			
Area_ijt_	Plot area (ha)	0.500	0.906
Collective_ijt_	1 = collective plot; 0 = no	0.875	0.331
Tenure_ijt_	1 = secure rights (customary or formal) over the plot; 0 = no	0.610	0.488
Intercropping_ijt_	1 = intercropping of legumes on the plot; 0 = no	0.181	0.384
Location_ijt_	1 = plot is located outside the household compound; 0 = no	0.375	0.484
Other production inputs			
Seed_ijt_	Seed applied (kg/ha)	35.12	16.63
Manure_ijt_	Manure applied (kg/ha)	7012	12,016
Herbicide_ijt_	Herbicide applied (cl/ha)	62.43	174.54
Fungicide_ijt_	Fungicide applied (g/ha)	8.87	79.67
Pesticide_ijt_	Pesticide applied (g/ha)	3.97	68.43
Raticide_ijt_	Raticide applied (g/ha)	4.30	49.93
Labor_ijt_	Number of adult labor days worked on plot (person days)	5.14	7.66
Plot manager characteristics			
Age_ijt_	Age of plot manager (years)	48.67	14.58
Head_ijt_	1 = plot manager is the household head; 0 = no	0.883	0.321
Credit_ijt_	1 = plot manager has had access to credit over the last 12 months; 0 = no	0.147	0.353
Extension_ijt_	Number of years since the plot manager has received any extension services (years).Top-coded at 5 years	4.66	0.982
Household characteristics			
Size_jt_	Number of people in the household (persons)	11.29	7.28
Livestock_jt_	Number of livestock owned by the household, measured in tropical livestock units (TLU)	8.15	19.52
Landholding_jt_	Total land cultivated by the household (ha)	3.83	4.58
Income_jt_	Value of non-farm household income (ln 000’ FCFA)	190	574
Cotton_jt_	Number of cotton hectares cultivated by household (ha)	0.633	2.20

Source: Authors, based on EPA data. Total *n* = 6701 maize plots

Yield, N and all covariates capturing land quality, plot characteristics, other production inputs, and plot manager characteristics vary across plots (i), households (j), and years (t). Rain and rain variability vary across household/village (j) and years (t). Household characteristics vary across households (j) and years (t). Soil types and agro-climatic zones vary across household/village (j)

Yield (Y) of maize grain was calculated in kg per ha based on the crop harvested as recalled by the plot manager and physical measurements of field plot area as recorded in the household survey data. The nitrogen (N) application rate is the N nutrient kilograms divided by the plot area (in ha). Total N nutrient kilograms were calculated by multiplying the quantities of NPK compound fertilizer and Urea by their N content (15 and 46%, respectively). As discussed above, agro-ecological conditions measured at several scales of analysis – field plot, village, and zone – were included. In addition to plot size, we controlled for whether the plot was collectively or individually managed,^6^ under secure land rights, and intercropped. Other productive inputs included whether manure, herbicide, fungicide, pesticide, or raticide were applied, the quantity of planted seeds, and labor used on the plot, as recalled by the plot manager and recorded in the household survey data. Whether the plot manager was the household head, had access to credit, and had received extension services were included along with his/her age. Household characteristics included household size, livestock, landholding, non-farm income, and cotton hectares. (See supplementary material for a summary table of timevariant variables across years).

Maize cultivation was more prominent in villages within the Sudanian zone, which accounted for approximately 4200 maize plots distributed across 166 villages. In contrast, there were about 5300 maize plots dispersed across 378 villages in the Sudano-Sahelian zone. The number of households using fertilizer rose from 38% in 2009/10 to 43% in 2010/11 and 48% in 2011/12. Although some households applied fertilizer discontinuously, over 80% used it each year over the three-year-period. Mean rates of N application at the plot level, including users and non-users, was 16 kg/ha. Not controlling for other covariates, the average maize grain yield without fertilizer use was ∼1100 kg/ha compared to ∼1475 kg/ha with fertilizer use. On average, both yields and rates of fertilizer application were higher in the Sudanian zone than in the Sudano-Sahelian zone (1389 kg/ha vs. 1148 kg/ha; 21 kg N/ha vs. 12 kg N/ha). In villages characterized with excellent soils for maize cultivation, average yields and application rates were 1213 kg/ha and 16 kg N/ha compared to 1180 kg/ha and 8 kg N/ha for villages with good soils.

## 4 Results and discussion

### 4.1 Maize yield response to fertilizer

Table 2 shows the CFA-CRE regression results for three different yield response functions. Model 1 included agro-ecological conditions at the field plot level only (land quality indicators). Model 2 included agro-ecological conditions at both plot and village levels, adding soil type and rainfall indicators. Model 3 included agro-ecological indicators measured at three scales of analysis, also controlling for zone. All models included a quadratic term for nitrogen and control for other productive inputs, plot, plot manager and household characteristics, in addition to time dummies and household time-averages.

**Table 2:** Maize yield function estimation results under different agro-ecological conditions

Variable	Model 1	Model 2	Model 3
N	25.82***	23.90***	20.95***
	(5.68)	(6.09)	(6.11)
N^2^	–0.0157***	–0.0217***	–0.0218***
	(0.0046)	(0.0052)	(0.0051)
Agro-ecological conditions			
SWC	82.91**	69.82**	77.63**
	(35.84)	(34.72)	(33.98)
Fallow	0.964	1.496*	1.536*
	(0.799)	(0.811)	(0.812)
Agroforestry	34.06	39.57	35.16
	(28.80)	(28.02)	(28.00)
Lowland	284.40***	309.91***	316.02***
	(60.48)	(63.16)	(61.88)
Slope	92.64*	90.04*	85.01*
	(51.50)	(50.20)	(50.30)
Rain		–0.536**	–0.698***
		(0.226)	(0.212)
CV		–678.22*	–759.61**
		(393.20)	(378.2)
Excellent soils		59.55**	54.10*
		(30.30)	(30.60)
Good soils		233.13***	235.84***
		(38.91)	(38.85)
Sudano-sahelian			–98.38*
			(51.45)
N*SWC	2.000*	2.220*	2.048*
	(1.203)	(1.317)	(1.257)
N*Fallow	–0.0196	–0.0191	–0.0199
	(0.0204)	(0.0210)	(0.0201)
N*Agroforestry	–1.887**	–1.625**	–1.470**
	(0.734)	(0.737)	(0.729)
N*Low	–1.698	–2.902**	–2.968**
	(1.387)	(1.432)	(1.414)
N*Slope	–0.873	–0.815	–0.641
	(1.522)	(1.401)	(1.405)
N*Rain		0.000765	0.003339
		(0.00240)	(0.00270)
N*CV		20.15***	19.81***
		(6.981)	(6.999)
N*Excellent soil		–2.225***	–2.143***
		(0.731)	(0.729)
N*Good soil		–3.201***	–3.423***
		(1.036)	(1.041)
N*Sudano-sahelian			1.505*
			(0.801)
Other production inputs			
Seed	–2.670***	–2.465***	–2.470***
	(0.893)	(0.867)	(0.868)
Manure	–0.00217**	–0.00228**	–0.00230**
	(0.00110)	(0.00113)	(0.00112)
Herbicide	–0.675***	–0.660***	–0.682***
	(0.241)	(0.235)	(0.228)
Fungicide	–0.638	–0.612	–0.642
	(0.406)	(0.398)	(0.395)
Pesticide	0.102	0.0645	0.0751
	(0.315)	(0.3531)	(0.3562)
Raticide	–0.465	–0.440	–0.444
	(0.283)	(0.269)	(0.274)
Labor	–1.594	–1.272	–1.307
	(1.891)	(1.773)	(1.790)
Plot characteristics			
Area	163.22***	170.53***	170.42***
	(23.73)	(25.58)	(25.66)
Location	–105.33**	–111.02***	–116.22***
	(41.98)	(42.33)	(41.65)
Collective	–116.71***	–119.90***	–117.31***
	(45.03)	(45.19)	(45.52)
Tenure	–11.65	1.599	–0.736
	(23.96)	(25.82)	(25.96)
Intercropping	–151.84***	–155.00***	–159.93***
	(38.29)	(40.08)	(40.87)
Plot manager characteristics			
Age	0.290	–0.213	–0.206
	(0.809)	(0.825)	(0.827)
Credit	–116.94**	–122.54**	–123.63**
	(57.81)	(57.02)	(57.06)
Head	103.12**	99.85**	97.43**
	(49.32)	(48.19)	(48.33)
Extension	33.17**	30.61*	30.44*
	(15.06)	(15.88)	(15.82)
Household characteristics			
Size	2.409	2.276	2.035
	(3.570)	(3.929)	(3.970)
Livestock	1.497	1.166	1.120
	(1.996)	(1.936)	(1.946)
Landholding	6.767	3.638	4.221
	(11.52)	(11.21)	(11.15)
Income	0.0810**	0.076*	0.0768*
	(0.0405)	(0.0421)	(0.0423)
Cotton	–32.14	–39.80*	–40.71*
	(19.99)	(20.02)	(21.07)

Source: Authors, based on EPA data. Total n = 6701 maize plots. *, **, *** significant at the 10%, 5%, and 1% levels, respectively. All models include a constant, time dummies, and household time-averages. Standard errors are bootstrapped

Models 1–3 controlled for endogeneity of nitrogen use. The F-statistic of the first-stage regression (shown in the supplementary material) was highly significant (F-statistic = 22.90; Prob > F = 0.0000)^7^ The instrument, cotton cooperative membership, was strongly correlated with the potentially endogenous variable, nitrogen application rate (coefficient = 10.62 with a *p* value = 0.000). The exclusion restriction was also highly plausible because the proportion of households belonging to a cotton cooperative at the commune level is unlikely to affect maize yields at the plot level in our econometric specification. Therefore, the instrument was considered to be reliable and valid. Based on the assumptions regarding the instrument, the null hypothesis that fertilizer is exogenous can be rejected. The predicted residual of the first stage regression estimating the nitrogen application rate was also highly significant (*p* value = 0.000) in the second stage regression of the CFA-CRE models.

As expected, the coefficient estimate of nitrogen application rate was positive and significant across the models, whereas its squared term was negative and significant, indicating that as nitrogen application rate increased, maize yield increased at a decreasing rate. As more agro-ecological conditions were included, the coefficient estimate of nitrogen application rate became smaller; from approximately 26 kg/ha in the shorter model (column 1) to 21 kg/ha in the full model (column 3). This corroborated the importance of agro-ecological conditions. Other coefficient estimates remained robust to the inclusion of more variables. Results of the full model (column 3) are further discussed.

The estimated kg of maize grain produced per kg of N applied (24.1) was in line with other estimates for maize based on data from farmers’ fields in Sub-Saharan Africa. In their review, Yanggen et al. (1998) found response rates to be less robust in West Africa than in East and Southern Africa, with some under 15, most in the 10–15 range, and few over 25 kg of output per kg of N. More recent meta-analyses of experimental studies include Vanlauwe et al. (2011), who reported maize grain increases of 17–26 kg harvested per kg N for maize hybrids, and even higher rates when manure or compost were mixed with mineral fertilizer. Only one of the studies analyzed was conducted in Burkina Faso, however, and it was not referenced. Comparing across cereals and various regions of the world, Ladha et al. (2005) reported a mean of 24 kg of additional maize grain per kg of N. Using cross-sectional farm household data, Koussoube and Nauges (2017) and Ragasa and Chapoto (2017) estimated that one kg of nitrogen per ha led to maize yield increases of 19 kg per ha in Burkina Faso and between 22 and 26 kg per ha in Ghana. Estimated maize yield responses to nitrogen in Kenya are considerably higher. In Western Kenya, Marenya and Barrett (2009) estimated that one kg of N per ha led to maize yield increases of 40–44 kg per ha and emphasized the heterogeneity in profitability among farms in their sample. Sheahan et al. (2013) reported an overall increase of 17 kg of maize grain per kg of N in Kenya, but with great variation among agro-ecological zones, ranging from 11 kg to 39 kg. They found the highest response rate in the least fertile zone where fertilizer use is lower and more recent. In Zambia, Xu et al. (2009) found response rates ranging from under 10 to over 30 kg of maize grain, with a median of 16 kg of maize grain produced per kg of N applied.

Most of the agro-ecological conditions at the plot level, which were expressed in land quality indicators, were statistically significant at the 10% level or lower. The marginal effect^8^ of lowland plots on maize yields was positive. With no application of N, lowland plots have significantly higher yields than those on higher ground. In Burkina Faso, lowland areas are among the most fertile since they benefit from the application of fertilizer upstream (GIZ 2012), residual moisture and soil deposits. This is supported by the interaction term, which indicated that lowland plots benefited the least from an additional kg of N. Fallowing of land also supports crop yields. The presence of anti-erosion structures (SWC) also positively affected maize yields. This is consistent with previous research which showed that farmers cultivating in agro-ecological zones characterized by low rainfall and soil fertility, as in many parts of Burkina Faso, have higher incentives to adopt these practices in order to increase both land quality and crop productivity (Sawadogo and Kini 2011).

Introducing agro-ecological conditions at the village level, we found positive marginal effects of good and excellent soil types on maize yields. In the absence of nitrogen application, yields were significantly higher on good and excellent soils compared to poor or marginal soils. Interaction terms between N application and soil types were also statistically significant, with poor and marginal soils benefiting the most from an additional kg of N. Likewise, Burke et al. (2017) estimated lower yields on clayish and sandy soils in Zambia. Using farmers’ perceptions of soil quality rather than soil types, Koussoube and Nauges (2017) and Ragasa and Chapoto (2017) did not find a statistically significant relationship between soil quality and maize yields. Moreover, the marginal effect of rain and rainfall variability on yield was negative. As discussed by Koo and Cox (2014), the variability of total rainfall during the maize growing season is lower in regions characterized by limited water availability. In our results, without N application, yields were significantly higher in villages characterized by less variability in rainfall and smaller amounts of rain. This may be explained by the fact that it is the availability of moisture over the cropping season, rather than the amount of rainfall, that most determines yields. The interaction term of rainfall variability was also statistically significant, with the benefit from an additional kg of N being greater in villages that experienced greater rainfall variability.

After controlling for agro-ecological conditions at the field plot and village levels, a statistically significant yield differential was found between the Sudano-Sahelian and Sudanian zones. The interaction term between N application and agro-ecological zone was also statistically significant, with maize production benefiting the most from an additional kg of N in the Sudano-Sahelian zone. The marginal effect of the Sudano-Sahelian zone on maize yield was negative. In comparison, Ragasa and Chapoto (2017) found lower yields in the Northern Savannah zone of Ghana and hypothesized that it was due to poorer soils and erratic rainfall patterns than the southern zones, but the interaction term was not significant.

Like Guirkinger et al. (2015) in neighboring Mali, we found lower yields on plots that were collectively managed compared to those individually managed on maize, which is a high value crop in Burkina Faso. Intercropping, which often involves cultivating maize with a legume, such as groundnut or cowpea, on the same plot during the same growing season, negatively affected maize yields. Yields for a single crop on intercropped plots often appear to be low because the areas cultivated with that crop are overstated; the overall economic return on that plot could be higher because of the other crops. Plots that were located farther away from the residence had significantly lower yields, perhaps because they are more difficult to reach with manure and fertilizers.

Among the productive inputs, the coefficient of herbicide use was negative and significant, although its use was limited. Unlike fertilizer, this yield-protecting input does not increase the potential yields but instead reduces the potential yield losses, since it is applied to prevent or respond to a weed infestation. The unexpected negative sign of the manure coefficient may reflect that the amounts of manure applied may have supplied insufficient nutrients to support the growth of maize plants. Compared to inorganic fertilizers, nutrients in manure are less concentrated and become available over a period of months or years rather than in a few days to weeks (Whiting et al. 2014). In the long-run, the application of manure to agricultural fields should improve soil conditions through an increase in soil organic matter, and in turn yields. The coefficient of the quantity of seeds planted was also significant and negative. This may indicate that some farmers had to replant seeds, delaying germination and thereby, leading to lower yields. This practice is often observed in the region.

Some plot manager characteristics do affect maize yield response to N. Maize plots managed by household heads had significantly higher yields, probably reflecting the role of the head in management of production and grain stocks for the family as a whole (Kazianga and Wahhaj 2013). Having access to credit last year and more recent contact with extension services were negatively associated with maize yields. Likewise, Xu et al. (2009) found that farmers receiving advice from extension agents can be less likely to adopt organic manure and thus, their yields are lower. These findings should be interpreted carefully, since these are only proxies for plot manager characteristics. They do not indicate that credit and extension services are detrimental to productivity gains, since we do not know for sure whether credit was used for maize production and whether extension services were orientated toward maize productivity. They do indicate that plot manager characteristics influence yields and that there is reason to control for them in yield response functions.

### 4.2 Profitability of fertilizer use

In Burkina Faso, the Ministry of Agriculture and Food Security recommends 100 kg/ha of urea and 150 kg/ha of NPK compound fertilizer on maize, regardless of the agro-ecological conditions (MA/SG 2001). The uniform recommended rate across all agro-ecological conditions is equivalent to 68.5 N nutrient kg/ha. Our data show that the quantity of N applied to field plots (conditional on use) falls short nationwide, with an average of 38 kg/ha. The gap is even more striking when we consider plots with no fertilizer applied, reducing the nationwide average to 16 kg N/ha.

Based on the yield function estimates of the full model, the estimated average grain yield increase per ha for each kg of N applied per ha at the sample means across all agro-ecological conditions was 24.1 kg. However, as seen in Table 3, the average partial effects of N vary across agro-ecological conditions measured at macro and micro scales. For instance, the yield response to fertilizer on a lowland plot with good soils in the Sudano-Sahelian zone averaged 18 kg/ha compared to 23 kg/ha for a lowland plot with marginal/poor soils in the Sudanian zone. More particularly, the presence of soil and water conservation structures raised the yield response on marginal or poor soils.

**Table 3:** Yield response to fertilizer by soil types and land quality indicators in the Sudano-Sahelian and Sudanian zones

	Sudano-sahelian zone			Sudanian zone		
	Lowland	SWC	Agroforestry	Lowland	SWC	Agroforestry
Excellent soils	19.14	23.74	21.34	20.65	25.24	22.85
Good soils	17.86	22.46	20.06	19.37	23.96	21.57
Poor/marginal soils	21.28	25.88	23.50	22.79	27.38	24.99

Source: Computed by the authors. Note: The average partial effects are computed by setting the variables of interest at 1, with everything else set at their sample means

**Fig. 1:**
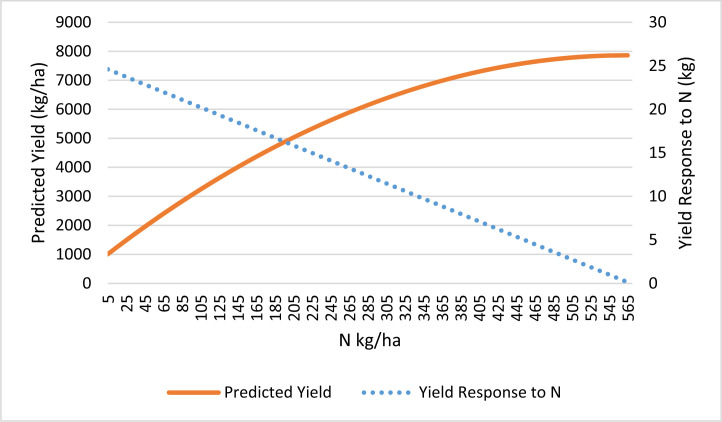
Predicted maize yield and yield response to nitrogen application in Burkina Faso. Source: Authors, based on EPA data

Figure 1 shows the predicted yield and yield response variations with respect to nitrogen application rate. On average, the N application that attains the highest grain yield possible without other limitation is estimated at about 500 kg N per ha, which far exceeds the actual and recommended application rates. Our estimate is higher than previously estimated in Burkina Faso (78 N kg/ha, by Koussoube and Nauges 2017), but compares favorably with other studies that were based on farm household survey data. For instance, in Ghana, Ragasa and Chapoto (2017) estimated an ‘agronomic’ optimum of 250 N kg/ha. This is expected, since Ghana is a country characterized by better agro-ecological conditions for maize production than Burkina Faso. The higher value of our estimate is driven primarily by the small magnitude of the estimated coefficient for N-squared (–0.022) in the yield response function. However, the magnitude of the estimated coefficient for N-squared is similar to that estimated in previous studies based on farm household survey data.^9^ Our result is consistent with evidence that shows continuous depletion of soil fertility in this region (see Stoorvogel et al. 1993; World Bank 1996 cited by Gruhn et al. 2000; Henao et al. 1992).

Table 4 reports the MVCRs and AVCRs under different scenarios, using the expected marginal product (24.1 kg/ha) and expected average product (24.5 kg/ha) attributable to N, respectively. These are expected values calculated at variable means, although the VCR does vary with position on the fertilizer response curve. Given the nature of farming in Burkina Faso, where crops depend entirely on rainfall, there is uncertainty in regard to the outcome of fertilizer use. At market prices, the expected MVCRs are mostly below two, indicating that income would not increase with an increase in fertilizer use. However, with the subsidies, plot managers could increase their income by applying more fertilizer, as evidenced by the MVCRs above two. At full market prices, expected AVCRs are below two, indicating that fertilizer use is unprofitable at low and average farm gate prices for maize. The ratios rise above three with an official price subsidy of 50%, despite low farm gate prices for maize. At the official subsidized price, incentives to use fertilizer are strong (above three), overcoming price and production risks. Transaction costs erode the apparent advantages of the subsidy, however (ratios above two but below three). Often, in addition to the poor condition of roads and transport distances, illicit taxation and bribery are reported (Holtzman et al. 2013).

**Table 4:** Marginal and Average Value-Cost Ratio under Various Prices

Scenarios	Fertilizer at market price		Subsidized fertilizer price		Subsidized fertilizer price + transaction costs	
	MVCR	AVCR	MVCR	AVCR	MVCR	AVCR
Low maize price	1.7	1.8	3.5	3.5	2.3	2.4
Average maize price	1.9	1.9	3.8	4.8	2.5	2.6
High maize price	2.1	2.1	4.2	4.3	2.8	2.9

Source: Computed by the authors. Note: Low, average, and high maize farm-gate prices are 123 FCFA/kg, 134 FCFA/kg, and 149 FCFA/kg, respectively. The value-cost ratios are computed by setting the variables of interest at 1, with everything else set at their sample means

The economically optimal rates of N under differing agro-ecological conditions are presented in Table 5. Those estimates are based on expected MVCRs of two, full market prices for fertilizer, and average farm-gate prices for maize. As seen, application rates vary widely across agro-ecological conditions, reaching 133 kg/ha of N in lowland plots with good soils in the Sudano-Sahelian zone to over 300 kg/ha of N on plots, where soil and water conservation practices are applied, in the Sudanian zone. These rates are higher than those previously estimated by Koussoube and Nauges in Burkina Faso (65–119 kg/ha of N) but similar to those in Ghana (130–256 kg/ha of N). Taken together, these findings show that fertilizer application rates, and thereby, profitability of fertilizer use vary across agro-ecological conditions, even for plots located in the same agro-ecological zones.

**Table 5:** Economically optimal N rates under Differing Agro-Ecological Conditions

	Sudano-Sahelian zone			Sudanian zone		
	Lowland	SWC	Agroforestry	Lowland	SWC	Agroforestry
Excellent soils	163	268	213	197	302	248
Good soils	133	239	184	168	273	218
Poor/marginal soils	212	317	262	246	351	297

Source: Computed by the authors. The rates are calculated using expected marginal value-cost ratios of 2 and by setting the variables of interest at 1, with everything else set at their sample means

In Burkina Faso, the adjusted net national income per capita was approximately 236,000 CFA over the three-year-period of the survey (World Bank 2018).^10^ Given that one additional kg of N can increase maize yields by 24 kg/ha, the use of an additional bag of 50 kg of NPK fertilizer can lead to an increase in maize yields of 180 kg/ha, which corresponds to a 14% increase in maize yields. Assuming that maize is sold at the average national price and that fertilizer is acquired at the subsidy price, this maize yield increase translates into an additional income of 11,620 CFA or a 5% income increase.

## 5 Summary and conclusions

Intensification strategies that aim to boost productivity while protecting natural resources have become central to agricultural growth and food security, especially in the West African Sahel, where land resources are becoming increasingly limited and population pressures heavy. For the most part, agricultural policies have emphasized the use of inorganic fertilizer through subsidy programs and with blanket recommendations nationwide. Yet, little is known about how agro-ecological conditions on farms affect fertilizer use and profitability. This study contributes to the sparse literature on maize yield responses to fertilizer in West Africa by uniquely combining information from a household farm panel with rainfall and soil data in order to study agro-ecological conditions at three different scales (field plot, village, and zone) in Burkina Faso.

Using a Control Function Approach with Correlated Random Effects, we estimated a grain yield increase of 24.1 kg per ha for each kg of N applied per ha, which is within the range reported in the few comparable studies conducted in the region. Maize crop productivity, as well as the marginal effect of N, differed significantly according to agro-ecological conditions. Several agro-ecological characteristics of field plots proved to be important for maize productivity and marginal product of fertilizer, including the presence of soil and water conservation structures and location of the field in the lowlands, where nutrients and moisture more readily accumulate. Likewise, soil types and rainfall variables measured at the village level significantly influenced both maize yield and its response to fertilizer. Our finding also shows that agro-ecological zone matters. Maize yield was significantly lower in the Sudano-Sahelian zone, after controlling for other agro-ecological conditions at the plot and village levels. Overall, these results highlight the importance of agro-ecological conditions, especially at the local scale, and the need to reformulate fertilizer recommendations that better take them into consideration. Micro-variations in agro-ecological conditions at the village and field plot levels appear to be more critical in our results than agro-ecological zone definition.

As expected in an uncertain farming environment with poorly developed markets for fertilizer, plot managers apply fertilizer at lower than the profit-maximizing rate. We found that while fertilizer use is mostly unprofitable at full market prices, it was, on average, profitable if we assumed that farmers benefit fully from the 50% fertilizer subsidy in place in Burkina Faso. Transaction costs diminish the benefits of the subsidy, and we know these to be widespread and household-specific.

Our findings have important policy implications, supporting research from field trials with analysis of farm household survey data. For example, policy makers need to be cautious when generalizing across regions or drawing policy recommendations from a single agro-ecological zone because crop responses and economic incentives vary widely across agro-ecological conditions. Policies that take heterogeneity into account may be more effective in promoting sustainable input use by making it more profitable, although the cost effectiveness of such programs would need to be assessed since these are expected to be harder to design and costlier to implement. As currently designed, the fertilizer subsidy program promotes maize, which is not well-suited to all agro-ecologies in Burkina Faso. Programs targeted to a single crop may not be desirable, especially in the context of climate change. Although the subsidy enhances profitability (to the extent that it covers transaction costs), there may be more effective ways to make fertilizer more affordable to farmers. For instance, investing in road infrastructure and removing illicit tax collection could lead to significant cuts in transaction costs while freeing up resources from the agricultural budget to enable other services, such as research and development and extension. With respect to future research, more concerted efforts to integrate methods utilized in agronomic analysis of field trials with household survey data could provide greater insights. In the meantime, recent analyses based on household survey data are relatively uncommon in the West African Sahel.

## Footnotes

1 Several instrumental variables were tested, including the proportion of households that belong to cotton cooperatives at the village and commune levels, the proportion of households that have had access to credit the previous year at the village level, and the median prices of fertilizer at the commune level. Due to the limited number of observations, fertilizer price was a poor predictor of actual fertilizer application rates. To avoid problems associated with weak instruments, we did not use fertilizer price as an instrument. Among the other three IVs, the proportion of households that belong to cotton cooperatives at the commune level was the strongest. Note that the instrument excludes the village where the household lives.

2 E(MVCR_ijt_) = E(MP_ijt_) * P_maize_/P_N_.

3 Like Xu et al. (2009), we use the price of urea and NPK and their nutrient content to estimate the price of nitrogen per kilogram. The amount of each fertilizer required for 1 kg is given by 0.46X + 0.15X = 1. Solving for X, we get X = 1.63. Therefore, 1 kg of nitrogen costs approximately 1.63 kg of each fertilizer, or P_N_ = 1.63 (P_urea_ + P_NPK_)

4 E(AVCR_ijt_) = E(AP_ijt_) * (P_maize_ /P_N_)

5 Less than 1% of maize plots were located in the Sahelian zone (below 600 mm isohyet).

6 In the dryland farming systems ofthe West African Sahel, farms operated by extended families headed most often by a senior male patriarch continue to prevail. These farming households are typically composed of members who are vertically (e.g., married sons and their wives) and horizontally (e.g., brothers and multiple wives) linked to the household head. The head is responsible for managing plots farmed collectively to serve the overall starchy staple needs of the household and for allocating individually fields among household members to meet their personal needs and constitute a household “reserve.” (e.g, Guirkinger et al. 2015; Kazianga and Wahhaj 2013).

7 The F-statistic was nearly 23, which is much larger than the Staiger and Stock (1997) rule of thumb (value of 10). The F-statistic is also considerably larger than the Stock-Yogo critical value for a 10% level of statistical significance (16.38), suggesting that weak instruments are not a concern. Therefore, robust inferences to test for weak instruments are not necessary.

8 The marginal effect ofa covariate (e.g., lowland) takes into consideration the individual effect of the covariate (lowland) as well as its interacted effect (lowland*N) on yields.

9 Ragasa and Chapoto (2017) estimated a N-squared coefficient of –0.04 in Ghana; Burke et al. (2017) reported coefficients of –0.004 and 0.002 for basal and top dress fertilizer in Zambia, respectively. Koussoube and Nauges (2017) calculated coefficients ranging from –0.03 to –0.11. They estimated the fertilizer application rate that maximizes yields using the N^2^ coefficient of –0.11 from the 3 SLS regression, which is greater in absolute term than our estimate and those presented in other studies. Our result would have been more similar to theirs had they used the estimated coefficient from the 3SLS-FE regression (–0.03).

10 US $1 roughly equals 500 CFA
